# Supplementary material for: Re-evaluating randomized clinical trials of psychological interventions: Impact of response shift on the interpretation of trial results
Source: PLoS One. 2021 May 25;16(5):e0252035. doi: 10.1371/journal.pone.0252035 (PMC8148324; doi:10.1371/journal.pone.0252035)
Supplement: S1 Appendix — (DOCX) [file pone.0252035.s001.docx]

**S1 Appendix: Detailed results of the response shift investigation per dataset**

Supplementary material to: Re-evaluating randomized clinical trials of psychological interventions: Impact of response shift on the interpretation of trial results. M.G.E Verdam, W. van Ballegooijen, C.J.M. Holtmaat, H. Knoop, J. Lancee, F.J. Oort, H. Riper, A. van Straten, I.M. Verdonck-de Leeuw, M. de Wit, T. van der Zweerde, M.A.G. Sprangers

**S1 Table. The decomposition of change for the items of the insomnia severity index (ISI) that was the primary outcome in the CBT for insomnia dataset.**

**S1 Fig. The multigroup measurement model of insomnia severity at baseline and follow-up assessment.**

**S2 Table. The decomposition of change for the subscales of personal meaning profile (PMP) that was the primary outcome in the personal meaning for cancer survivors dataset.**

**S2 Fig.** **The multigroup measurement model of personal meaning at baseline and follow-up assessment.**

**S3 Table. The decomposition of change for the subscales of the centre for epidemiological studies depression scale (CES-D) that was the primary outcome in the CBT for depressive symptoms in patients with diabetes dataset.**

**S3 Fig. The multigroup measurement model of depression at baseline and follow-up assessment.**

**S4 Table. The items per subscales of the personal meaning profile (PMP)**

**S5 Table. The items per subscales of the centre for epidemiological studies depression scale (CES-D).**

**S1 Table. The decomposition of change (Cohen’s d [95% confidence interval]) for the items of the insomnia severity index (ISI) that was the primary outcome in the CBT for insomnia dataset.**

|  | **CBT group** | | **Control group** | |
| --- | --- | --- | --- | --- |
| **Item** | **Observed change** | **Response shift** | **Observed change** | **Response shift** |
| 1. Difficulty falling asleep | -0.94  [-1.24; -0.63] |  | -0.34  [-0.47; 0.04] |  |
| 1. Difficulty staying asleep | -1.77  [-2.01; -1.54] | -0.75  [-0.99; -0.52] | -0.25  [-0.48; -0.03] |  |
| 1. Problems waking up too early | -0.80  [-1.14; -0.47] |  | -0.19  [-0.42; 0.12] |  |
| 1. Satisfaction with sleep pattern | -1.76  [-2.01; -1.51] |  | -0.46  [-0.64; -0.29] |  |
| 1. Interference daily functioning | -1.57  [-1.78; -1.35] |  | -0.41  [-0.59; -0.23] |  |
| 1. Noticeable impact on QoL by others | -1.01  [-1.24; -0.78] |  | -0.30  [-0.50; -0.11] |  |
| 1. Worries about sleep problems | -1.64  [-1.88; -1.40] |  | -0.42  [-0.63; -0.21] |  |
| Change in the underlying target construct, i.e. insomnia severity | -1.75  [-2.00; -1.51] |  | -0.58  [0.78; -0.38] |  |


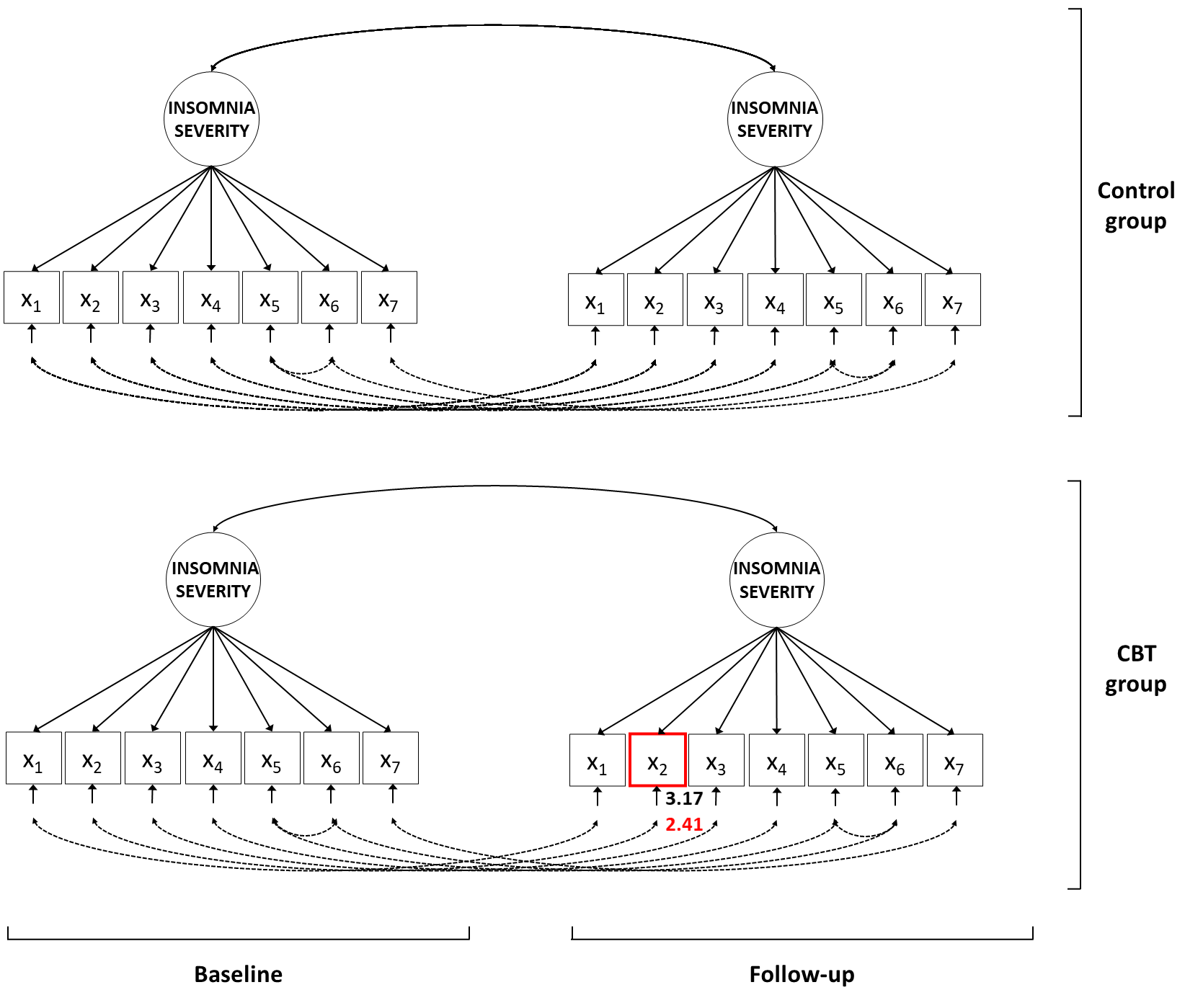


**S1 Fig. The multigroup measurement model of insomnia severity at baseline and follow-up assessment.**

*Notes*: The squares represent the observed item-scores (X) of the insomnia severity index measured at both baseline and follow-up assessment. The solid single-headed arrows at the bottom represent the residual factors of each item. The dotted double-headed arrow represents the relations between the residual factors, where the residual factors of the same item are allowed to correlate over time. The circles represent the construct that the items aim to measure (i.e., insomnia severity, both at baseline and follow-up assessment). Each arrow from a circle to an item represents a factor loading. The double-headed arrows between the circles represent the correlations between insomnia severity over time. Note that the model includes a residual correlation between the observed item-scores X_5_ and X_6_. This modification makes sense given that both items ask about interference of sleep problems, an aspect that is not specifically addressed in the other items of the ISI (see Table 1). Recalibration response shift was detected for X_2_ in the CBT-group only; the numbers depicted at the bottom of X_2_ represent the intercept values at baseline (black) and follow-up (red) assessment respectively.

**S2 Table. The decomposition of change (Cohen’s d [95% confidence interval]) for the subscales of personal meaning profile (PMP) that was the primary outcome in the personal meaning for cancer survivors dataset.**

|  | **MCGP-CS group** | | **Control group** | |
| --- | --- | --- | --- | --- |
| **Subscale** | **Observed change** | **Response shift** | **Observed change** | **Response shift** |
| Relation with God/higher order (RG) | 0.12  [-0.10; 0.35] | 0.05  [-0.18; 0.27] | -0.12  [-0.31; 0.07] | 0.06  [-0.13; 0.25] |
| Dedication to life (DL) | 0.21  [0.06; 0.37] |  | -0.22  [-0.37; -0.06] |  |
| Fairness of life (FL) | 0.12  [-0.08; 0.32] |  | -0.14  [-0.32; 0.02] |  |
| Goal-orientedness (GO) | 0.20  [0.01; 0.39] |  | -0.25  [-0.53; 0.02] |  |
| Relations with other people (RO) | 0.14  [-0.04; 0.33] |  | -0.11  [-0.32; 0.10] |  |
| Change in the underlying target construct, i.e. personal meaning | 0.52  [0.46; 0.58] |  | -0.50  [-0.59; -0.41] |  |


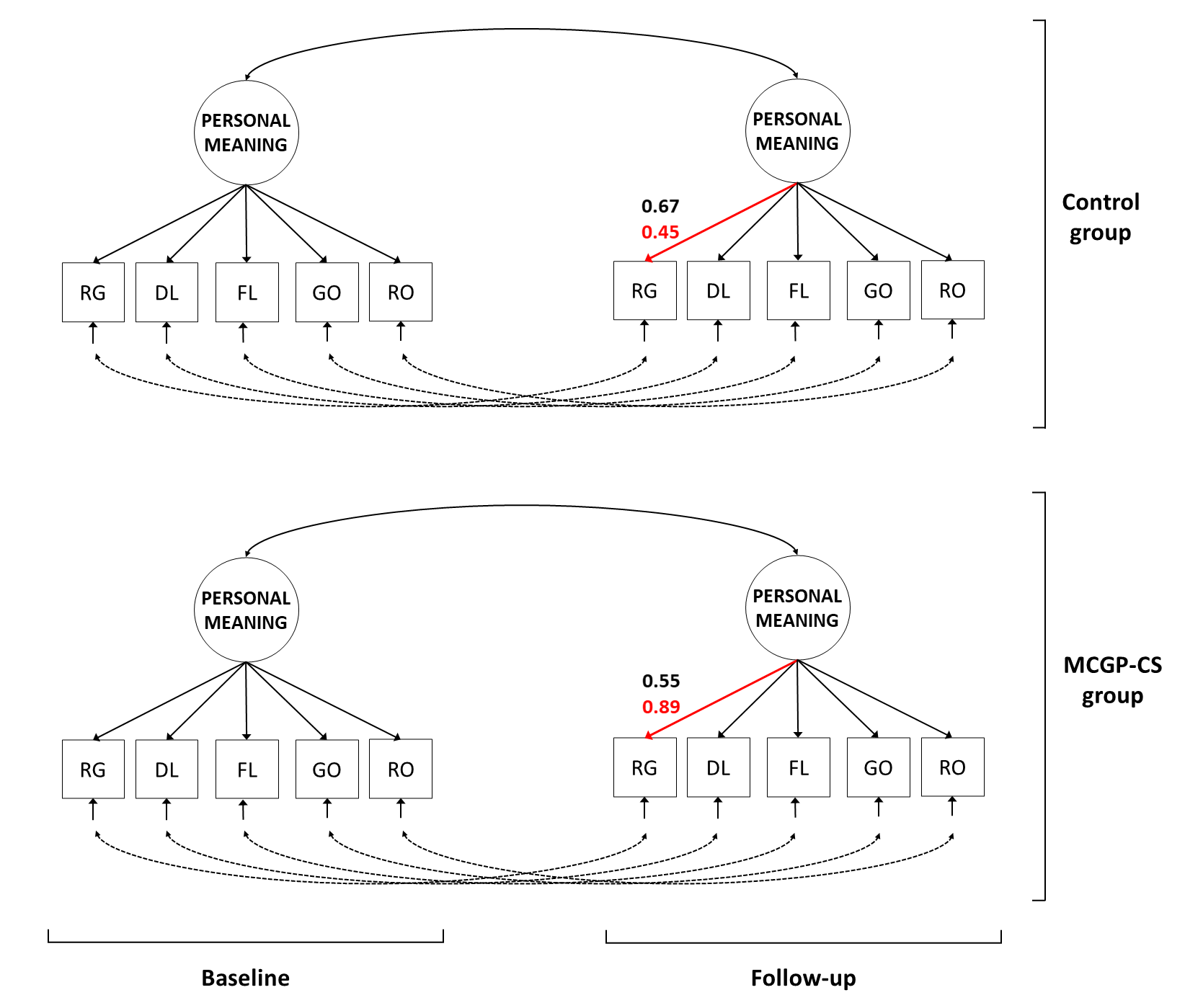


**S2 Fig. The multigroup measurement model of personal meaning at baseline and follow-up assessment.**

*Notes*: The squares represent the observed scale-scores of the personal meaning profile (Dutch version) measured at both baseline and follow-up assessment: relation to God/higher order (RG), dedication to life (DL), fairness of life (FL), goal orientation (GO), and relations with others (RO). The solid single-headed arrows at the bottom represent the residual factors of each subscale. The dotted double-headed arrow represents the relations between the residual factors, where the residual factors of the same subscale are allowed to correlate over time. The circles represent the construct that the items aim to measure (i.e., personal meaning, both at baseline and follow-up assessment). Each arrow from a circle to an item represents a factor loading. The double-headed arrows between the circles represent the correlations between personal meaning over time. Reprioritization response shift of RG was detected in both control and MCGP-CS groups; the numbers depicted represent the values of the (unstandardized) factor loadings at baseline (black) and follow-up (red) assessment respectively.

**S3 Table. The decomposition of change (Cohen’s d [95% confidence interval]) for the subscales of the centre for epidemiological studies depression scale (CES-D) that was the primary outcome in the CBT for depressive symptoms in patients with diabetes dataset.**

|  | **CBT group** | | |  | | **Control group** | | | |
| --- | --- | --- | --- | --- | --- | --- | --- | --- | --- |
| **Subscale** | **Observed change** | | **Response shift** | | | **Observed change** | | **Response shift** | |
| Well-being (WB) | -0.97  [-1.15; -0.79] | |  | | | -0.44  [-0.56; -0.32] | |  | |
| Depressed affect (DA) | -1.17  [-1.31; -1.02] | | 0.96  [0.81; 1.10] | | | -0.71  [-0.82; -0.59] | | 0.46  [0.35; 0.57] | |
| Somatic symptoms (SS) | -0.12  [-1.25; -0.98] | |  | | | -0.52  [-0.61; -0.43] | |  | |
| Interpersonal problems (IP) | -0.69  [-0.83; -0.54] | |  | | | -0.28  [-0.40; -0.17] | |  | |
| Change in the underlying target construct, i.e. depression | -1.58  [-1.77; -1.38] | |  | | | -0.80  [-1.00; -0.61] | |  | |
|  |  |  | | |  | |  | |  |
|  |  |  | | |  | |  | |  |


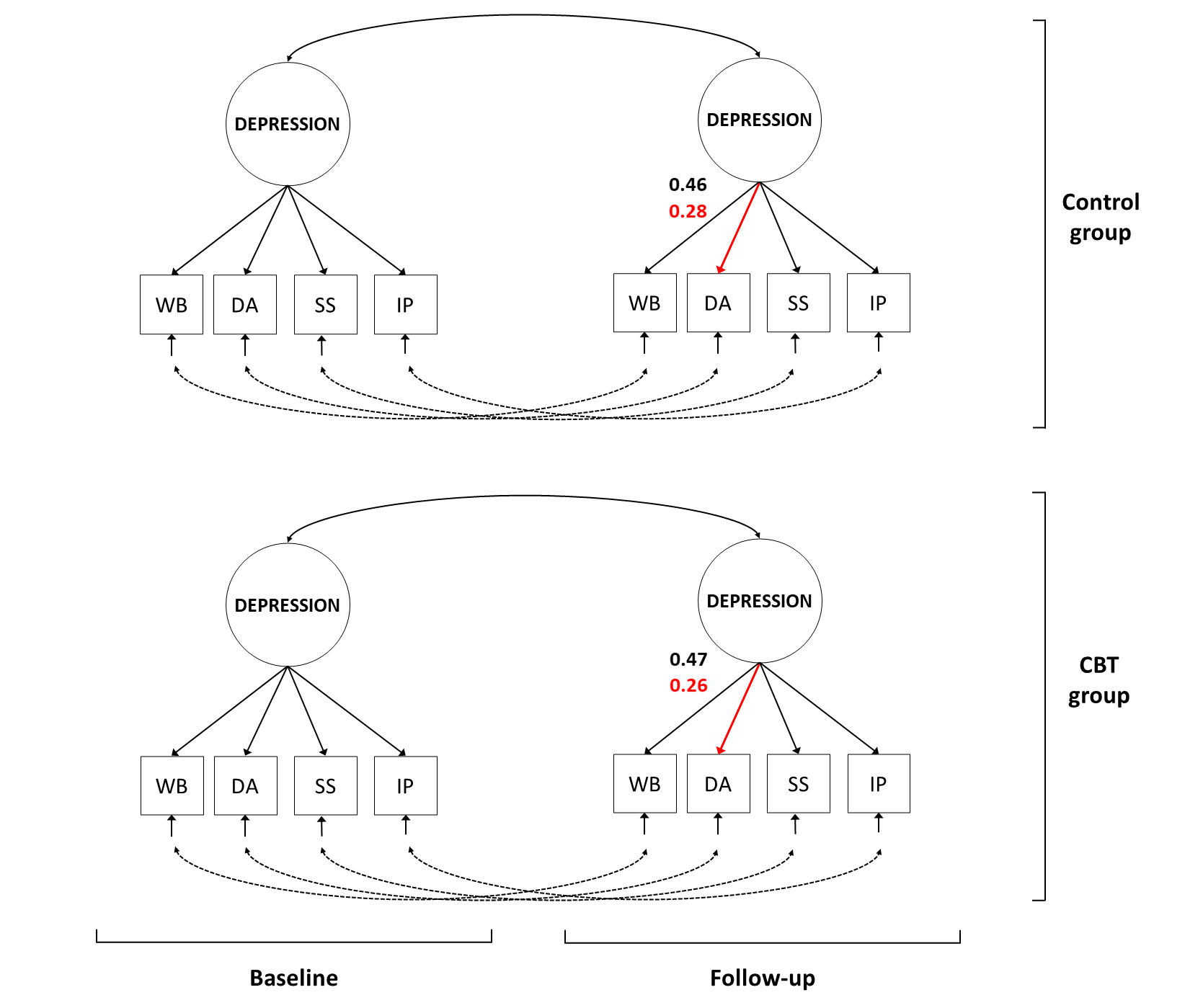


**S3 Fig. The multigroup measurement model of depression at baseline and follow-up assessment.**

*Notes*: The squares represent the observed scale-scores of the CES-D measured at both baseline and follow-up assessment: wellbeing (WB), depressive affect (DA), somatic symptoms (SS), and interpersonal problems (IP). The solid single-headed arrows at the bottom represent the residual factors of each subscale. The dotted double-headed arrow represents the relations between the residual factors, where the residual factors of the same subscale are allowed to correlate over time. The circles represent the construct that the items aim to measure (i.e., depression, both at baseline and follow-up assessment). Each arrow from a circle to an item represents a factor loading. The double-headed arrows between the circles represent the correlations between depression over time. Reprioritization response shift of DA was detected in both control and CBT groups; the numbers depicted represent the values of the (unstandardized) factor loadings at baseline (black) and follow-up (red) assessment respectively.

**S4 Table. The items per subscales of the personal meaning profile (PMP)**

| **Relation with God/higher order (RG)** |
| --- |
| Peace with God |
| Believe in afterlife |
| Seek to do God’s will |
| Can have a personal relationship with God |
| Seek to glorify God |
| Sense of mission or calling |
| Believe there is order and purpose in the universe |
| Seek higher values |
| **Dedication to life (DL)** |
| Contribute to the well-being of others |
| I value my work |
| Make a significant contribution to society |
| Committed to my work |
| I take initiative |
| I like challenge |
| Persistent and resourceful in attaining my goals |
| Able to make full use of my abilities |
| Strive toward personal growth |
| Do not give up by setbacks or obstacles |
| I am altruistic and helpful |
| **Fairness of life (FL)** |
| Life has treated me fairly |
| I am at peace with my past |
| I accept my limitations |
| Received fair share of opportunities and rewards |
| There is rough justice in this world |
| I accept what cannot be changed |
| I am treated fairly by others |
| I am at peace with myself |
| **Goal-orientedness (GO)** |
| I strive to achieve my life goals |
| I pursue worthwhile objectives |
| I believe in the value of my pursuit |
| Life has an ultimate purpose and meaning |
| I have a purpose and direction in life |
| I seek to actualize my potentials |
| **Relations with other people (RO)** |
| I have a mutually satisfying relationship |
| I have found someone I love deeply |
| I have someone to share intimate feelings with |
| I have a good family life |
| I have confidants to give me emotional support |
| I relate well to others |
|  |

| **S5 Table. The items per subscales of the centre for epidemiological studies depression scale (CES-D).** |
| --- |
| **Well-neing (WB)** |
| Just as good as others |
| Hopeful about the future |
| Happy |
| Enjoyed life |
| **Depressed affect (DA)** |
| Can't shake the blues |
| Felt depressed |
| Life has been a failure |
| Fearful |
| Lonely |
| Crying spells |
| Sad |
| **Somatic symptoms (SS)** |
| Bothered by things |
| Appetite was poor |
| Trouble concentrating |
| Everything was an effort |
| Sleep was restless |
| Talked less than usual |
| Could not get going |
| **Interpersonal problems (IP)** |
| People were unfriendly |
| People disliked me |
